# Virginia’s inpatient mental healthcare geography post SB260

**DOI:** 10.1016/j.hpopen.2025.100152

**Published:** 2025-11-17

**Authors:** Mary Schwoerer, Timothy F. Leslie

**Affiliations:** Geography and Geoinformation Science, George Mason University, Fairfax, USA

**Keywords:** Mental health policy, Psychiatric hospital utilization, Medicaid access

## Abstract

This study explores the impact of mental health policy reforms on geographic variations in inpatient psychiatric facility utilization and mental health outcomes in Virginia. Following the enactment of Senate Bill 260 (SB260), we observed significant changes in utilization patterns, particularly in regions with higher proportions of Medicaid-eligible populations. We identify nuanced factors influencing facility usage, including proximity to facilities and demographic characteristics, shedding light on the complex dynamics of mental health care access. Notably, our analysis indicates a notable increase in overall utilization of Virginia’s state-operated mental hospitals post-SB260, suggesting a greater fulfillment of unmet needs for inpatient care. Moreover, our research underscores the necessity to reconsider IMD exclusion laws, emphasizing the potential benefits of policy changes for underserved populations. This research contributes to mental health policy discussions by offering evidence-based considerations for future reforms aimed at improving access and equity in mental health care delivery in Virginia.

## 1 Introduction

The complex spectrum of mental health care ranges from non-medical psychological counseling to residential inpatient treatment, and patients have a wide range of ability to integrate into and function in society. Prior to the mid-20th century, centralized state-operated mental institutions were the standard option for individuals unable to function independently in society due to their mental conditions, which could include intellectual disability and mental illness, as well as severe physical impairments. These institutions were wrought with abuse and poor living conditions, and there was a widespread effort to close mental institutions in favor of decentralized community-based services with care better tailored to the patients’ specific needs. In many cases, the institutions were closed without sufficient services being stood up to take their places. The availability of local services varied widely from a geographic perspective, and in some cases, the results of “deinstitutionalization” were the creation of homeless populations living in proximity to wherever they could access services[1]. Mentally ill individuals also ended up in prison[2], committing suicide[3], and many other unintended negative consequences[4,5].

The challenges stemming from the closure of centralized mental institutions underscored the need for tailored solutions to address mental health crises at the community level. This need became evident in November 2013 when a tragic incident involving the son of a Virginia State Senator shed light on deficiencies in the state's mental health system. Unable to detain the individual under a Temporary Detention Order due to a lack of available psychiatric beds, regional health authorities were forced to release him while in a state of mental crisis, leading to devastating consequences[6]. This event served as a catalyst for legislative action, culminating in the passage of Virginia State Bill 260 (SB260), which enacted significant reforms aimed at bolstering the state's mental health care infrastructure. SB260 established key reforms including: (1) creation of the Virginia Psychiatric Bed Registry providing real-time bed availability, (2) extension of temporary detention orders from 48 to 72 h, (3) designation of state hospitals as 'beds of last resort,' and (4) standardized regional admissions protocols. These SB260 reforms were expected to increase access and utilization through several mechanisms. The Virginia Psychiatric Bed Registry was designed to reduce placement delays by providing real-time visibility of available beds, thereby decreasing the likelihood of patients being released due to inability to locate appropriate facilities. The extension of temporary detention orders from 48 to 72 h provided additional time for clinicians to stabilize patients and secure appropriate placements. Most critically, the 'beds of last resort' provision required state hospitals to accept patients when community beds were unavailable, creating a safety net that would capture previously unmet demand. Together, these reforms aimed to address the bed shortage crisis that led to Gus Deeds' release by establishing both improved coordination mechanisms and capacity guarantees, which we hypothesize would manifest as measurable increases in state facility utilization, particularly among underserved populations.

The State of Virginia runs 7 geographically distributed inpatient mental health facilities, excluding two specialty facilities serving children and adolescents and geriatric patients. For adults not institutionalized by the criminal justice system, these facilities augment private mental health institutions or community-based services for individuals requiring inpatient hospitalization [7]. Since Virginia implemented mental health reforms in 2014, the state facilities are also required to take patients admitted under Temporary Detention Orders when alternative beds at local community hospitals cannot be found for acute care. Another reform was creating an on-line psychiatric bed registry to simplify the process of placing patients. Comparing facility use before and after this legislation provides a unique opportunity to measure the impact of policy on the mental health care system by describing regional utilization patterns of the state inpatient mental health institutions in the State of Virginia and analyze changes in those patterns after the implementation of new legislation in the summer of 2014.

Despite SB260′s implementation over a decade ago, research on its geographic impacts remains limited, especially for vulnerable populations facing access barriers. This study delves into the impact of these reforms on the utilization patterns of Virginia's inpatient mental health facilities. By comparing facility use before and after the legislative changes, the analysis aims to reveal regional disparities in mental health care access. Examining U.S. deinstitutionalization outcomes challenges the assumption that community-based services adequately replaced state institutions. In particular, this study addresses two primary questions: (1) What is the relationship between geographic access to mental health services, state facility usage, and population mental health outcomes? (2) How has psychiatric facility utilization evolved following SB260 implementation? The investigation will also assess the relationship between access to alternative services, hospital utilization, and indicators of community mental health.

## 2 Materials and Methods

### 2.1 Methods

Understanding the relationship between geographic access to mental health services and population outcomes necessitates a multivariate approach to evaluating access metrics. This study incorporates several key metrics: the distance to state-operated and community-based mental health facilities, the availability of outpatient mental health clinics accepting Medicaid, mental health provider ratios, and geographic variables such as altitude. Distance-based measures were chosen based on established 'Jarvis Law' literature demonstrating distance decay in facility utilization[8]. Access to healthcare is influenced by the supply of services, their appropriateness, affordability, and physical accessibility[9]. Travel times between mental health facilities were estimated using population-weighted centroids of census tracts to assess accessibility accurately. Aligning with the United States Department of Health and Human Services Health Resources and Service Administration (HRSA) methodology for identifying Health Provider Shortage Areas (HPSAs), the study evaluates mental health provider ratios by considering the number of providers per capita and their distribution within counties, as reported in the Robert Wood Johnson Foundation’s County Health Rankings[10]. Furthermore, the distance a patient must travel to access mental health services, a crucial factor influencing service utilization[11], is a significant consideration. Altitude, as a metric for topographical challenges, offers additional insights into access barriers that conventional measures may overlook. Mental health outpatient clinics accepting Medicaid were used as proxies for access to community-based services, a critical factor in regression modeling given the shift from institutional care to community-based services under deinstitutionalization policies[12]. A correlation analysis was conducted to explore demographic factors influencing access levels, building on national studies that identified significant relationships between access disparities and demographic factors such as race, poverty, and rurality[12]. This study employs a hybrid methodology combining container-based (e.g., Transit performance score, staffed psychiatric beds, mental health provider rate) and distance-based measures (e.g., drivetime to state-operated inpatient hospitals, other inpatient facilities, federally qualified health centers, community mental health centers, and additional facilities from the National Directory of Mental Health Treatment) to describe mental health care access at both the county and Community Services Board (CSB) levels, utilizing Ordinary Least Squares (OLS) models with R statistical software to examine linear relationships between access variables and outcomes while controlling for demographic confounders. Following established geographic health research standards, this study reports p-values and standardized coefficients to assess statistical significance and effect magnitude. While confidence intervals provide additional precision estimates, the geographic nature of this analysis emphasizes spatial relationships and policy impact significance, consistent with mental health geography literature[13].

To assess temporal changes in utilization rates between 2013 and 2015, we employed a matched-pairs *t*-test comparing utilization rates for each of the 40 CSBs at both time points. This paired analysis treats each CSB as its own control, comparing pre-SB260 (2013) and post-SB260 (2015) utilization for the same geographic unit. The matched-pairs approach accounts for individual CSB characteristics and baseline utilization levels, providing a more rigorous assessment of whether SB260 implementation resulted in statistically significant changes in state facility usage. The null hypothesis tested was that the mean difference in utilization rates between time points equals zero, with significance assessed using a two-tailed test.

The study focuses on several primary mental health outcome measures to understand the impacts of geographic access to mental health services and legislative changes. These include state mental facility utilization rates, suicide rates, self-reported mentally unhealthy days from surveys, and discussions on mental health on social media platforms. State mental facility utilization rates were assessed using data from Virginia's Department of Behavioral Health and Developmental Services, which provided detailed information on bed-days utilized across the state's nine psychiatric inpatient facilities. To establish correlations with mental health outcomes, suicide rates were obtained from the Virginia Department of Health, covering data from 2003 to 2008, and Pearson's correlation analysis was applied. Additionally, survey data on self-reported mentally unhealthy days was sourced from the Behavioral Risk Factor Surveillance System (BRFSS) and reported by County Health Rankings, encompassing data periods from 2005 to 2012. This study also explores the potential of using social media data to predict regional mental health care outcomes by comparing variations in mental health discussions against traditional measures.

Regression analysis stands at the forefront of our investigation, aiming to unveil the intricate relationship between geographic access to mental health services and consequential health outcomes. Employing an Ordinary Least Squares (OLS) approach, our models delve into the impact of various access metrics on critical mental health indicators, with a particular focus on state mental facility utilization rates and suicide rates. We incorporate a range of independent variables to comprehensively capture the multifaceted nature of access to mental health care. Central to our analysis is the assessment of distance to facilities, which signifies the travel time from population-weighted centroids of census tracts to the nearest mental health treatment facilities. This includes state-operated inpatient hospitals, community mental health centers, and other pertinent service providers. Recognizing the distinct access challenges encountered in rural locales compared to urban counterparts, we integrate a measure of rurality into our models. Additionally, we explore the documented correlation between altitude and depression, as evidenced by seminal studies[14,15,16,17], by examining the association between the average altitude of counties and suicide rates. Availability of services is gauged through crucial metrics such as the density of mental health providers per capita and the presence of outpatient clinics accepting Medicaid, providing insight into the accessibility of mental health services. To address accessibility disparities among populations without personal vehicles, we incorporate the transit performance score as a pivotal variable. Through this comprehensive approach, our regression models aim to elucidate the interplay between geographic access to mental health services and consequential health outcomes, shedding light on critical determinants of mental health care utilization and accessibility.

To gauge the importance of mental health facilities within specific service areas and understand patient behavior, we introduce two key metrics: the Relevance Index (RI) and the Commitment Index (CI). The RI measures the proportion of individuals from a Community Services Board (CSB) region who utilized a given hospital, providing insights into the facility's significance within the CSB's catchment area. The use of RI and CI is valuable in identifying bypass behavior and prompting further investigation into the reasons and likelihood of a population not utilizing their most proximal facility[[18], [19]]. The Relevance Index of each hospital to a population with utilization from a given CSB is calculated as follows:

$${\mathrm{RI}}_{x,csb}=\frac{U_{x,csb}}{U_{\mathrm{csb}}}$$

Where ${\mathrm{RI}}_{x,csb}$ is the proportion of individuals from a CSB region who utilized a given hospital. $U_{x,csb}$ is the utilization of a given hospital (x) by individuals from a given CSB and $U_{\mathrm{csb}}$ is the total utilization of all hospitals from that CSB. Conversely, the CI assesses the proportion of individuals utilizing a hospital that originated from a particular CSB, shedding light on patient referral patterns and the facility's attractiveness to different markets. The Commitment Index for each hospital to determine where the majority of its patience originate is calculated as follows:

$${\mathrm{CI}}_{x,csb}=\frac{U_{x,csb}}{U_{x}}$$

Where ${\mathrm{CI}}_{x,csb}$ is the proportion of individuals who utilized a given hospital originated from a particular CSB, $U_{x,csb}$ is the utilization of a given hospital (x) by individuals from a given CSB and $U_{x}$ is the total utilization of that hospital.

Analyzing changes in facility utilization post-SB260 entails a multifaceted approach. Firstly, we calculate changes in patient travel distances by multiplying hospital bed days by average travel time and dividing by total bed days from each CSB. This calculation provides insights into shifts in patient access to mental health services following the implementation of SB260. Furthermore, changes in RI and CI from 2013 to 2015 are computed for each region, offering a comprehensive understanding of evolving utilization patterns and facility importance over time.

To ascertain the statistical significance of changes in utilization rates, we employ a matched pairs test. This test compares utilization rates between two time points (pre- and post-SB260) for each CSB region, accounting for individual CSB characteristics and potential confounding factors. By rigorously assessing changes in utilization rates, we can discern the true impact of SB260 on mental health facility utilization across Virginia.

### 2.2 Materials

This study primarily focuses on the county level within Virginia, with exceptions for state facility utilization data available at the Community Services Board (CSB) scale. County-level data is aggregated into Virginia's 40 CSBs to ensure consistency and eliminate the influence of varying policies on mental health care outcomes. Virginia's unique trend of increased utilization of state-operated mental institutions post the enactment of SB-260′s Bed of Last Resort legislation contrasts with the national trend of decreasing state facility utilization rates since 1970. Previous research in mental health geography, often conducted at the county level, aligns with this study's focus. In Virginia, mental health services are organized through CSBs, comprising adjoining counties or independent cities, emphasizing the significance of county-level analysis.

Data sources and the study variables derived from them are summarized in Table 1 below.

**Table 1:** 

Data Category	Data Description	Source
Outcome	Bed-days and Originating CSBs for Virginia’s inpatient adult psychiatric hospitals	Virginia Department of Behavioral Health
Outcome	Suicide and Homicide Rates	Virginia Violent Death Reporting System
Outcome	Average Mental Unhealthy Days	2013 County Health Rankings report
		2015 County Health Rankings report
Access	Facility Information	National Directory of Mental Health Treatment Facilities
		Federally Qualified Health Centers
Access	Drivetimes	ESRI global drivetimes
Access	Transit Performance Score	Center for Neighborhood Technology
Access	Altitude	USGS Digital Elevation Model,
	1/3 arc-second DEM	
Access	Staffed Psychiatric Beds	2013 Richmond times article (*Psychiatric Beds in Virginia*, 2013) reported staffed beds cross checked with AHRF field f1143717 from American Hospital Association data, NDMHTF
Access	Mental Health Provider Rate	2015 County Health Rankings Report
Population Data	Medicaid Eligible Population	f1420010 Area Health Resource File 2010 Medicaid Eligible Population Ages 21–64 from Centers for Medicare & Medicaid Services (CMS.gov)
Population Data	Reported Excess Alcohol	2013 County Health Rankings (from 2005 to 2011 Behavior Risk Factor Surveillance System data)
Population Data	Uninsured	2013 and 2015 County Health Rankings
Population Data	Demographic, Education, and Income Data	2013 County Health Rankings
		2010 TIGER/Line® Shapefiles and Area Health Resource File
Administrative Boundaries	Census.gov	2010 TIGER/Line® Shapefiles

The study utilizes data from the Virginia Department of Behavioral Health and Developmental Services (DBHDS) regarding the state's nine state-operated psychiatric inpatient facilities. This data includes facility addresses and the number of bed-days utilized. Additionally, utilization rates for non-state-operated facilities are derived from the American Hospital Association (AHA) and incorporated from the Area Health Resource File (AHRF) dataset provided by the Health Resources and Services Administration (HRSA). However, it is important to note that the AHA data lacks detailed information on patient origins, which limits its applicability to this analysis. The DBHDS publishes detailed reports on the utilization of state mental health (MH) facilities, categorized by various demographic groups such as children and adolescents, civil adults, forensic adults, and geriatric patients. This data is further organized by Health Planning Region (HPR) and Case Management CSB. For this study, the focus is on civil adult usage, with data normalized by the 2010 population to determine utilization rates in terms of hospital bed days per 1,000 residents aged 18–64. To evaluate changes in state-operated psychiatric inpatient utilization before and after the 2014 legislative reforms (SB-260), facility utilization data from 2013 and 2015, as reported by the Virginia DBHDS, is analyzed.

Suicide rates for the period from 2003 to 2008 were obtained from the Virginia Department of Health's Office of the Chief Medical Examiner, as reported in the Virginia Violent Death Reporting System [20]. This dataset was chosen due to its pre-combined age-adjusted rates for each Community Services Board (CSB), facilitating streamlined analysis. Although the Virginia Department of Health's Statistical Reports and Tables also provide suicide counts and age-adjusted rates by county, the lack of county-level age distributions introduced significant aggregation errors, which precluded the use of more recent data from this source. Instead, annual age-adjusted rates were directly acquired from the Virginia Department of Health (VDH) website, offering county and statewide segmentation further divided by demographic categories[21].

Data on “poor mental health days” was sourced from the County Health Rankings (CHR) project, drawing on the Behavioral Risk Factor Surveillance System (BRFSS). The 2013 CHR report covered data from 2005 to 2011, while the 2015 CHR report encompassed data from 2006 to 2012. To ensure completeness of the dataset, any missing values were addressed by averaging data from both reports for counties with available information in both periods. For counties with data only from one report, the available value was utilized. Out of 135 counties, 24 lacked data, predominantly low-population counties where privacy concerns and statistical significance pose challenges. For the CSB dataset, population-weighted averages of mentally unhealthy days from constituent counties were calculated. In cases where any counties within a CSB lacked data, the average value from the remaining counties was used to maintain the integrity of the dataset.

To evaluate patient travel time to mental health treatment facilities, we calculated travel times from the geographic centroid of each census tract to the nearest available facility. A population-weighted mean distance was employed to estimate the average patient travel time from each Community Services Board (CSB) to the respective facilities. This involved calculating the distance (in time) from each county to each facility using the network center and then weighting this by the county population before averaging across the CSB population. This methodology was similarly applied at the census tract scale prior to aggregating the data to the CSB level. In addition to drive times, Transit Performance Scores obtained from the Center for Neighborhood Technology were incorporated to account for accessibility for populations without personal vehicles. Facility addresses and service types were sourced from SAMHSA’s 2016 National Directory of Mental Health Treatment Facilities and HRSA.gov. For travel time calculations, census tract attribute data was transformed to the geographic centroid of each area using a table join. When needed, population centroids were aggregated into county or CSB-level data through a population-weighted centroid calculation.

To explore the relationship between suicide rates in Virginia and altitude, we used average elevation data congruent with the health outcome reporting scale to enhance correlation robustness. The county dataset in HRSA’s Area Health Resource File (AHRF) relies on elevation data from a 1977 System Sciences Study, which is incomplete in some instances. In these cases, we supplemented with average county elevations derived from 1/3 arc-second Digital Elevation Models provided by the USGS. This dual approach ensures a comprehensive dataset for examining the potential influence of altitude on mental health outcomes, particularly suicide rates.

Prior to the enactment of SB260, comprehensive data on staffed and available psychiatric hospital beds in Virginia was sparse. Currently, the Virginia Psychiatric Bed Registry, established under SB260, provides such information to authorized users, enhancing transparency and access. However, for the period preceding SB260′s enactment, estimates of available psychiatric beds were essential. The study leveraged data from a 2013 report by the Richmond Times, which was cross-referenced with the Area Health Resource File (AHRF) for accuracy. This cross-referenced data, encompassing figures for both licensed and staffed beds, was deemed the most comprehensive and reliable for the time frame under consideration[22]; Richmond [23]. This approach enabled a critical historical perspective on the availability of psychiatric beds before the legislative changes brought by SB260.

The mental health provider rates for counties, obtained from the Robert Wood Johnson County Health Rankings, quantify the availability of mental health providers per capita. This metric is derived from National Provider Identifier (NPI) data available through the National Plan and Provider Enumeration System (NPPES). The metric's container-based nature, using providers per county instead of a distance-based catchment area, reflects a common approach in public health data analysis. Although the scholarly debate on the efficacy of container-based metrics versus distance-based catchments persists, especially in the context of mental health services[24,25,26], using catchments is unlikely to enhance fidelity in this context. This is due to the discrepancy often observed between the registered and practice addresses of providers, particularly in public mental health services. Notably, administrative boundaries significantly influence service provision, as evidenced by residency requirements outlined by the Arlington County Community Services Board for accessing local mental health care (Arlington [27].

This analysis focuses on the Commonwealth of Virginia, employing counties and Community Services Boards (CSB) as the primary scales of investigation. The Virginia Department of Behavioral Health & Developmental Services (DBHDS) serves as a key data source, providing insights into the utilization of Virginia's state mental health institutions by CSBs. CSBs are regional entities comprising counties within the state of Virginia, responsible for administering mental health care for their respective constituencies.

To delineate the study area and administrative boundaries, the study utilized the 2010 US Census TIGERline files sourced from ESRI. These files, coupled with census tract-level data from the 2010 US Census, facilitated the creation of population-weighted centroids for each Virginia county. Subsequently, this county-level data underwent further aggregation to determine the population-weighted center of each CSB region. The geographic centroid of each county was then matched with the population-weighted geographic centroid of the respective CSB, culminating in the establishment of the overall demographic landscape. Additionally, all Geographic Information System (GIS) data not originally in Lambert Conformal Conic Virginia projection were transformed into it for consistency.

The geocoding process for mental health facilities involved utilizing Google Maps to obtain geographical coordinates since the National Directory of Mental Health Treatment Facilities (NDMHTF) provides addresses rather than geospatial files. The facilities were categorized into four main groups, with an additional “other” category accommodating facilities that did not neatly fit into these primary classifications. The four main categories are as follows: (i) Community Mental Health Centers Accepting Medicaid, (ii) Community Primary Care Centers Accepting Medicaid (FQHC), (iii) State Mental Health Facilities, and (iv) Public and Private Hospitals with Psychiatric Beds. This categorization provides a structured framework for the subsequent spatial analysis of mental health facility distribution and accessibility within the Commonwealth of Virginia.

## 3 Results

The initial mental health outcome variables included state facility utilization rates for 2013 and 2015, average suicide rates, average homicide rates, and average reported mentally unhealthy days. Comprehensive correlation matrices examining relationships between all study variables are provided in Appendix Table A1 (CSB scale) and Table A2 (County scale). Homicide rates showed no correlation with mental health outcomes and were excluded due to their close ties to race and education. Correlations between mental health care access and homicide rates were positive, suggesting that areas with more services had sociodemographic variables associated with crime. This finding reinforces the distinction between mental illness and crime, supporting calls to destigmatize mental health issues. The analysis confirmed that facility utilization and suicide rates were the most relevant dependent variables for quantifying mental health outcomes in this study. The BRFSS survey variable was introduced as an independent variable to assess its impact on the models.

Numerous OLS regression models assessed the impact of independent variables on suicide rates at the county and CSB scale, as well as state-operated inpatient facility utilization at the CSB scale, summarized in Table 2, Table 3, respectively. Table 2 presents four suicide prediction models that systematically test geographic scale effects and data source contributions. Models 1 and 2 use county-level data to establish baseline relationships, with Model 1 testing whether self-reported mental health days (BRFSS) predict suicide rates and Model 2 isolating objective access and demographic predictors alone. Models 3 and 4 replicate this structure at the CSB scale, allowing direct comparison of how geographic aggregation affects model performance and variable significance*.* Models 1 and 3 include BRFSS self-reported mentally unhealthy days as a predictor, while Models 2 and 4 exclude this variable to assess the impact of objective access and demographic measures alone. Models 1–2 use county-level data (N = 110–133), while Models 3–4 aggregate to the CSB scale (N = 40), allowing comparison of geographic scale effects on model performance. The percentage of African American race showed a negative correlation with both facility utilization (−0.4 for 2013 and −0.3 for 2015) and suicide rates (−0.33 for county and −0.4 for CSB), consistent with existing literature indicating lower suicide rates among African Americans. However, the study highlights the likelihood of mental health crises in African Americans leading to law enforcement engagement rather than medical treatment, contributing to disparities in mental healthcare access. The negative Beta for African American race raises questions about law enforcement's role in addressing mental health crises in this community, warranting further research [28,29,30,31]. Additionally, the study suggests exploring if law enforcement-related deaths offset the apparent lower suicide risk for African Americans, potentially explaining the negative Beta in suicide risk models.

**Table 2:** Suicide Prediction Models at CSB and County Scale.

	Model 1 − County Suicide Model including BRFSS data	Model 2 − County Suicide Model excluding BRFSS data	Model 3 − CSB Suicide Model including BRFSS data	Model 4 − CSB Suicide Model excluding BRFSS data
*Variable*	*Estimate*	*Estimate*	*Estimate*	*Estimate*
Intercept	22.4633**	31.3596***	0.8173	
Average Mentally Unhealthy Days	1.35523***		1.1281**	7.9760
Transit Performance				
Nearest State Facility Drivetime	−0.01354			0.0300*
Nearest Inpatient Facility Drivetime				
Nearest FQHC Drivetime	0.04212	0.0937**	0.1215**	0.1471**
Nearest CMHC Drivetime	−0.03441			
Nearest Other Facility Drivetime		−0.0274	−0.0375*	−0.0395*
Nearest MHTF			0.3920***	0.3558**
Mean Elevation	−0.005854*	−0.0036		
Staffed Psychiatric Beds				
Mental Health Provider Rate			0.0193**	0.0260***
Medicaid Eligible Population		0.6184**	0.6840**	0.9454***
Reported Excess Alcohol			0.1328	
Uninsured Population (2015)			0.0641	
Percent Female	−0.36251**	−0.4498**	−0.4442	−0.5416*
Percent Hispanic	−0.17419*	−0.1756*	0.0587	
Percent African American	−0.25774***	−0.2308***	−0.0954**	−0.1138***
Percent Rural			−0.0317*	−0.0341*
Percent Aged Under 18	0.19257		−0.2596*	−0.3262*
Percent Aged Over 65	0.19326	0.0.1339	0.2039	0.1423
Log Median Income Z score	−0.9978			1.1156
High School Graduation Rate	−1.1416**	−1.2072**	0.1905	0.1867
Percent Unemployment	0.7523*	0.6932**	0.8272**	1.4052**
N	110	133	40	40
Adj R-squared	0.6008	0.4765	0.9279	0.9120
Moran’s I (residuals)	−0.0625	−0.0933	−0.0457	−0.0004

Significance Codes: (***).001, (**).01, (*).05.

**Table 3:** State Facility Utilization Models.

	Model 5 – 2013 State Utilization	Model 6 – 2015 State Facility Utilization Model including BRFSS data	Model 7––2015 State Facility Utilization Model Access Variables Only	Model 7a – State Facility Utilization Model Access Variables with Spatial Lag	Model 8–––2015 State Facility Utilization Model Socio-demo-graphic Variables Only	Model 8–––2015 State Facility Utilization Model Socio-demo-graphic Variables Only	Model 9–––2015 State Facility Utilization Model excluding BRFSS data
*Variable*	*Estimate*	*Estimate*	*Estimate*	*Estimate*	*Estimate*	*Estimate*	*Estimate*
Intercept	3.3107		31.9408	4.8553*	2.9170	1.3307	4.9046
Average Mentally Unhealthy Days			−1.3380				
Nearest State Facility Drivetime				−0.0430	−0.0253		
Nearest Inpatient Facility Drivetime	−0.1181†		−0.0821†	−0.0010	−0.0064		
Nearest FQHC Drivetime				0.0038	−0.0412		
Nearest CMHC Drivetime	0.2130			−0.2256	−0.1396		
Nearest Other Facility Drivetime				0.1141†	0.0638		
Nearest MHTF	−0.4633		−0.3446†				
Mean Elevation	00.31		0.0050	0.0110*	0.0088**		0.0057
Staffed Psychiatric Beds			−0.0134				
Mental Health Provider Rate							
Medicaid Eligible Population	0.9581*		1.384**			1.0531**	0.8261*
Reported Excess Alcohol							
Uninsured Population (2015)							
Percent Female			−0.4821				
Percent Hispanic						0.0533	0.0637
Percent African American	−0.1969*		−0.1749**			−0.1513**	−0.1415**
Percent Rural						−0.0630*	−0.0559†
Percent Aged Under 18						−0.1419	−0.2607
Percent Aged Over 65	0.3440		0.2997			0.4834	0.3587
Log Median income Z score							
High School Graduation Rate							
Percent Unemployment							
Spatial Lag Coefficient					0.5197**		
N	40		40	40	40	40	40
Adj R-squaredR-squared (Model 6a only)	0.5151		0.6705	0.3217	0.6004	0.5858	0.6167
Moran’s I (residuals)	0.1540		0.0269	0.2307**		−0.125	−0.0764

Significance Codes: (**).001, (*).01, (†).05.

At the CSB scale, the most fitting model (Model 4) incorporating sociodemographic and access variables achieved an Adjusted R-squared value of 0.9120. Including survey values from the Behavioral Risk Factor Statistical Study raised this to 0.9279 (Model 3). However, at the county scale, these values decreased to 0.4765 (Model 2) and 0.6008 (Model 1), respectively. Suicide rates showed significant relationships with both access and sociodemographic variables. Drivetimes to FQHCs and the nearest MHTF played predominant roles in access-related variation in CSB models. Interestingly, higher mental health provider rates were associated with higher suicide rates, warranting further exploration into this relationship, potentially indicating service direction to areas with greater need.

Population-related variables at the CSB scale associated with increased suicide rates included the percentage of Medicaid-eligible population and unemployment, while variables linked to reduced risk included female sex, African American race, rural population, and proportion of population under 18. The slightly negative coefficient for rural populations may be due to collinearity with access-related variables. At the county scale, elevation emerged as significant from the access variable set, exerting a slight negative impact. In both county-scale models, unemployment contributed to increased suicide rates. Additionally, Medicaid eligibility was identified as a contributor in the non-BRFSS model. Population variables associated with lower suicide rates included female sex, Hispanic and African American race, and high school graduation rates. Incorporating survey data from the SAMSHA BRFSS survey enhanced suicide prediction models at both scales.

Table 3 examines state facility utilization through multiple analytical lenses. Model 5 (2013) and Model 6 (2015) compare pre- and post-SB260 patterns using full variable sets to assess temporal policy effects. Model 7 isolates access variables to quantify geographic barriers, while Model 7a adds spatial autocorrelation to test for regional clustering. Model 8 examines demographic drivers alone, and Model 9 provides the comprehensive 2015 model excluding BRFSS data. This staged approach separates access effects from demographic confounders and reveals how SB260 altered the predictive power of different variable classes. Model 5 (2013 pre-SB260) and Model 6 (2015 post-SB260) include both access and demographic variables to assess temporal changes. Models 7 and 8 isolate access-only and demographic-only effects respectively for 2015 data. Model 7a adds a spatial lag coefficient to test for geographic clustering effects. The 2013 model showed an adjusted R-squared of 0.5151, which improved to 0.6705 for the 2015 rates, suggesting SB260′s changes to access successfully addressed unmet population needs, leading to more accurate utilization rate reflections.

At the 0.05 significance level, the primary contributors to facility utilization rates for access variables in 2015 were the nearest inpatient facility drivetime and the nearest mental health treatment facility overall. Utilization at state facilities in 2015 was higher in areas further away from other types of services. Conversely, in 2013, the distance to inpatient facilities other than state-operated ones was the sole significant access variable at p < 0.05 and played a larger role overall. This suggests that patients closer to other facilities with inpatient beds were less likely to use state facilities. Significant sociodemographic variables included the percentage of Medicaid-eligible population and African American race.

In both the 2013 and 2015 datasets, Medicaid eligibility emerged as a significant factor, with its coefficient increasing between the two periods. This connection is attributed to IMD exclusion rules, directing Medicaid patients to state-funded facilities for inpatient stays. Medicaid eligibility remained the strongest utilization predictor in both 2013 (β = 0.96, p < 0.01) and 2015 (β = 1.38, p < 0.001). This persistent effect, with increasing magnitude post-SB260, suggests that exclusion laws systematically channel Medicaid patients toward state facilities. This policy-driven utilization pattern contradicts principles of patient-centered care and creates a two-tiered system where insurance status determines facility access. Our findings support the growing consensus among mental health advocates and policymakers that IMD exclusion repeal would reduce access disparities and enable Medicaid beneficiaries to access the full continuum of inpatient psychiatric care.

SB260′s 'beds of last resort' mandate effectively created a safety net that captured demand previously unmet due to bed shortages. The 12.7 % utilization increase suggests substantial latent demand existed prior to reform. However, the continuing strength of Medicaid eligibility as a utilization predictor indicates structural barriers remain beyond bed availability. Repealing IMD exclusion would allow Virginia to optimize its mental health delivery system by enabling patient placement based on clinical appropriateness rather than insurance restrictions. Changes in relevancy and commitment indices highlight suboptimal utilization patterns in certain CSBs. To assess temporal changes in utilization rates between 2013 and 2015, we employed a matched-pairs *t*-test comparing utilization rates for each CSB at both time points. This paired analysis accounts for individual CSB characteristics and baseline utilization levels, providing a more rigorous assessment of whether SB260 implementation resulted in statistically significant changes in state facility usage compared to unpaired cross-sectional comparisons. The results indicated a significant difference, with a p-value of 0.004426 for a two-tailed test, affirming that 2015 usage diverged from 2013. While some CSBs witnessed a reduction in utilization, the statewide trend demonstrated an overall increase of 12.7 percent.

Two models outlining the relationship between study variables and utilization in 2013 and 2015 are summarized in Table 4. Notably, the only significant changes were a negative correlation with initial utilization rates (−0.3) and driving distance to the 'other' category of treatment facility (0.39). The trend of increased utilization in regions with lower initial rates suggests that service expansion, marked by new bed openings, may have addressed previously underserved populations. This warrants further investigation into long-term trends and persistent disparities in utilization rates post-service reconfiguration. The significance of the Medicaid-eligible adult population was evident in both years, particularly in 2015. Given limited options for Medicaid recipients due to the IMD exclusion policy, which directs them to state resources, the correlation between increased state beds and Medicaid eligibility aligns with literature suggesting IMD exclusion hinders Medicaid patients from accessing care outside the state system. This supports the effectiveness of SB260 in addressing unmet population needs.

**Table 4:** Change in State Mental Facility Utilization Rates from 2013 to 2015.

	Model 9–––2015 Utilization All Variables	Model 10–2013 Utilization All Variables
*Variable*	*Estimate*	*Estimate*
Intercept	31.9408	3.3107
Average Mentally Unhealthy Days	−1.3380	
Nearest State Facility Drivetime		
Nearest Inpatient Facility Drivetime	−0.0821*	−0.1181*
Nearest FQHC Drivetime		
Nearest CMHC Drivetime		0.2130
Nearest Other Facility Drivetime		
Nearest MHTF	−0.3446*	−0.4633
Mean Elevation	0.0050	0.0041
Staffed Psychiatric Beds	−0.0134	
Mental Health Provider Rate		
Medicaid Eligible Population	1.384***	0.9581**
Reported Excess Alcohol		
Uninsured Population (2015)		
Female	−0.4821	
Hispanic		
African American	−0.1749***	−0.1969***
Rural		
Under 18		
Over 65	0.2997	0.3440
Log income Z score		
High School Graduation Rate		
Percent Unemployment		
N	40	40
Adj R-squared	0.6705	0.5151
Moran’s I (residuals)	0.0269	0.0154

Significance Codes: (***).001, (**).01, (*).

The transformation in Relevance and Commitment Indices reveals shifts in both overall bed day usage and patterns of facility utilization by patients from different CSB regions. Changes in the Relevance Index indicate regions where patients began using different hospitals. The top 5 changes involve shifts from Alexandria CSB to Western State Hospital, Allegheny Highlands CSB to Catawba, Henrico CSB to Central and Eastern, Middle-Peninsula and Northern Neck CSB to Eastern State, and Piedmont patients to Catawba. Changes in the Commitment Index highlight cases where specific hospital populations received patients from different locations. The top 5 shifts involve decreases in CI at Catawba from Blue Ridge CSB, SWVMHI from Highlands CSB, and Central from Middle Peninsula-Northern Neck, along with increases in CSH from District 19 and ESH from Western Tidewater CSB. It's important to note that changes in RI didn't always align with changes in CI. For example, while CSH saw a 10 % increase in patients from District 19, this was due to District 19′s overall rise in utilization rate rather than a shift in the hospital utilized by that CSB.

## 4 Discussion and Conclusions

Following SB260 implementation, Virginia experienced a statistically significant 12.7 % increase in state psychiatric facility utilization (p = 0.004426), with the strongest effects observed in regions with higher Medicaid-eligible populations. This finding suggests that SB260 may have addressed previously unmet inpatient mental health needs, particularly for Medicaid beneficiaries who face access barriers due to IMD exclusion policies. The improved model fit for 2015 utilization data (Adj R^2^ = 0.67) compared to 2013 (Adj R^2^ = 0.52) indicates that post-SB260 utilization patterns became slightly more predictable from access and demographic variables, suggesting the reforms created more rational care-seeking patterns aligned with population needs and service availability.

In the aftermath of SB260′s enactment, which mandated Virginia's state-operated psychiatric facilities to accept clients instead of transferring them due to space constraints, the landscape of mental health service utilization in the Commonwealth underwent notable shifts. This legislation, pivotal in addressing access challenges to mental health care, forms the cornerstone of our investigation into the dynamics of mental health service utilization in Virginia. As the state grapples with the complexities of ensuring equitable access to mental health care, particularly in underserved areas, understanding the impact of SB260 on service utilization rates is paramount. Our study seeks to elucidate the implications of SB260 on mental health service dynamics, shedding light on the evolving landscape of mental health care accessibility and utilization post-policy implementation[32]. By examining the trends in utilization rates and patterns across different demographic and geographic strata, we aim to provide insights that inform future policy decisions and interventions aimed at improving mental health care delivery in Virginia. This discussion aligns closely with the goals set forth in our introduction, focusing on the impact of SB260 on mental health service utilization and its broader implications for mental health care accessibility and equity. This is in spite of methodological challenges such as multiple datasets with varying time periods and geographic scales, which may introduce temporal and spatial misalignment. The DBHDS utilization data lacks detailed patient-level characteristics, limiting ability to control for clinical severity.

The exploration of Medicaid eligibility as a key determinant of mental health challenges within the population emerges as a significant finding in our study, resonating with broader discussions surrounding mental health policy and access to care[33,34,35,36]. Medicaid, serving as a crucial safety net for vulnerable populations, plays a pivotal role in facilitating access to mental health services for those who might otherwise face barriers to care[37]. Our analysis underscores the importance of addressing disparities in mental health care access among Medicaid-eligible individuals, highlighting the need for targeted interventions and policy reforms aimed at bolstering mental health care provision for this demographic[33,34,35,36]. By examining the intersection of Medicaid eligibility and mental health outcomes, our study contributes to the ongoing discourse on health equity and access to care, particularly in the context of mental health services.

The disparities in access to mental health services, particularly in rural and minority communities, represent a critical area of concern[12]. Contrary to national trends, we observed patterns in service availability and utilization across different demographic and geographic contexts within Virginia. While rural areas typically face challenges related to limited-service availability and longer travel times, our analysis suggests that certain regions with higher proportions of Hispanic and African American populations exhibit shorter travel times and better service ratios, underscoring the complexity of access barriers in diverse communities[12,38,39]. The lower suicide rates among African Americans compared to whites observed in our study align with existing literature, highlighting the need for further investigation into the factors contributing to these disparities[38,39]. However, recent trends indicate potential worsening mental health outcomes among African Americans, emphasizing the importance of ongoing monitoring and intervention efforts [40,41]. Our study underscores the need for targeted interventions aimed at addressing disparities in access to care and improving mental health outcomes among underserved communities, particularly those disproportionately affected by structural barriers to care.

This study has important limitations. Temporal misalignment exists across datasets: suicide data (2003–2008) predates SB260, while facility location data (2016) postdates our utilization period (2013–2015). Second, without a control group or comparison state, observed changes may reflect broader trends rather than SB260-specific effects. Finally, missing data from 24 counties and aggregation to CSB-level may obscure within-region variation. These constraints limit causal inference but do not diminish the descriptive value of documenting utilization changes following SB260 implementation.

One of the aims of our study was to investigate the relationship between patient distance from psychiatric facilities and utilization rates, a concept rooted in Jarvis' Law[13]. While prior research suggested a correlation between proximity to mental health facilities and service utilization, our analysis at the Community Services Board (CSB) level did not find a consistent association between facility utilization and proximity. However, regression models revealed a significant relationship between state facility use and proximity to non-state inpatient hospitals, particularly following the implementation of SB260 in 2014. This finding suggests the potential for system optimization and the importance of considering interactions between different types of mental health facilities in shaping utilization patterns. Further research is warranted to better understand the drivers of facility utilization and the impact of policy reforms on access to mental health services, particularly in the context of evolving healthcare delivery systems. This multi-scalar analytical approach, combining both container-based and distance-based access metrics with policy evaluation, represents a methodological strength that allows more comprehensive assessment of access barriers than either measure alone.

Our analysis also uncovered significant correlations between various indicators of mental health outcomes, including suicide rates, state mental facility utilization, and survey-reported mentally unhealthy days. While previous assumptions suggested that increased access to community-based mental health services would lead to reduced inpatient facility utilization, our findings challenge this notion. The integration of multiple data sources, including both objective outcomes (facility utilization, suicide) and subjective measures (BRFSS), strengthens our ability to capture the multi-dimensional nature of population mental health and provides direct policy relevance for decision makers considering IMD exclusion reforms. We did not find a direct quantitative relationship between access to community-based services and the utilization of state-operated facilities in Virginia. Instead, our results suggest that Medicaid eligibility and African American race consistently emerged as significant predictors of mental health outcomes across various models. These findings underscore the need for an understanding of the factors influencing mental health outcomes and the importance of addressing systemic disparities in access to care. As we strive to improve mental health services and outcomes, it is imperative to consider the complex interplay of individual, socioeconomic, and policy factors shaping the mental health landscape.

## CRediT authorship contribution statement

**Mary Schwoerer:** Writing – original draft, Methodology, Conceptualization. **Timothy F. Leslie:** Writing – review & editing, Writing – original draft, Supervision.

## Declaration of competing interest

The authors declare that they have no known competing financial interests or personal relationships that could have appeared to influence the work reported in this paper.

## Figures and Tables

**Table A1:** Census Block Pearson’s Correlation Matrix for Model Variables. Bolded values are significant with p < 0.05.

**Table A2:** County Level Pearson’s Correlation Matrix for Model Variables. Bolded values are significant with p < 0.05.
